# Integrative analysis of gene expression and DNA methylation through one‐class logistic regression machine learning identifies stemness features in medulloblastoma

**DOI:** 10.1002/1878-0261.12557

**Published:** 2019-08-18

**Authors:** Hao Lian, Yi‐Peng Han, Yu‐Chao Zhang, Yang Zhao, Shan Yan, Qi‐Feng Li, Bao‐Cheng Wang, Jia‐Jia Wang, Wei Meng, Jian Yang, Qin‐Hua Wang, Wei‐Wei Mao, Jie Ma

**Affiliations:** ^1^ Department of Pediatric Neurosurgery Xinhua Hospital Affiliated to Shanghai Jiao Tong University School of Medicine China; ^2^ Institute of Health Sciences Shanghai Institutes for Biological Sciences Chinese Academy of Sciences University of Chinese Academy of Sciences Shanghai China; ^3^ School of Life Science Fudan University Shanghai China; ^4^ Huamu Community Health Service Center Shanghai China

**Keywords:** connectivity map, machine‐learning methods, medulloblastoma, prognostic model, stemness, tumor immune environment

## Abstract

Most human cancers develop from stem and progenitor cell populations through the sequential accumulation of various genetic and epigenetic alterations. Cancer stem cells have been identified from medulloblastoma (MB), but a comprehensive understanding of MB stemness, including the interactions between the tumor immune microenvironment and MB stemness, is lacking. Here, we employed a trained stemness index model based on an existent one‐class logistic regression (OCLR) machine‐learning method to score MB samples; we then obtained two stemness indices, a gene expression‐based stemness index (mRNAsi) and a DNA methylation‐based stemness index (mDNAsi), to perform an integrated analysis of MB stemness in a cohort of primary cancer samples (*n* = 763). We observed an inverse trend between mRNAsi and mDNAsi for MB subgroup and metastatic status. By applying the univariable Cox regression analysis, we found that mRNAsi significantly correlated with overall survival (OS) for all MB patients, whereas mDNAsi had no significant association with OS for all MB patients. In addition, by combining the Lasso‐penalized Cox regression machine‐learning approach with univariate and multivariate Cox regression analyses, we identified a stemness‐related gene expression signature that accurately predicted survival in patients with Sonic hedgehog (SHH) MB. Furthermore, positive correlations between mRNAsi and prognostic copy number aberrations in SHH MB, including MYCN amplifications and GLI2 amplifications, were detected. Analyses of the immune microenvironment revealed unanticipated correlations of MB stemness with infiltrating immune cells. Lastly, using the Connectivity Map, we identified potential drugs targeting the MB stemness signature. Our findings based on stemness indices might advance the development of objective diagnostic tools for quantitating MB stemness and lead to novel biomarkers that predict the survival of patients with MB or the efficacy of strategies targeting MB stem cells.

**Abbreviations**
AUC: area under the curve
CMap: Connectivity Map
CNS: central nervous system
CSC: cancer stem cell
DEG: differentially expressed gene
GEO: Gene Expression Omnibus
HRs: hazard ratios
K‐M: Kaplan–Meier
MB: medulloblastoma
mDNAsi: DNA methylation‐based stemness index
MoA: mode of action
mRNAsi: gene expression‐based stemness index
NK: natural killer
OCLR: one‐class logistic regression
OS: overall survival
ROC: receiver operating characteristic
SHH: Sonic hedgehog
WHO: World Health Organization
WNT: wingless

## 1 Introduction

Medulloblastoma (MB) is the most commonly diagnosed embryonal tumor of the central nervous system (CNS) in children. Despite being initially characterized based on histological features, it is now clearly accepted that MB mainly comprises four distinct molecular subgroups: wingless (WNT)‐activated, Sonic hedgehog (SHH)‐activated, group 3, and group 4, as reflected in the 2016 World Health Organization (WHO) classification of tumors of the CNS (Louis *et al*., 2016; Ramaswamy *et al*., 2016a,b). These four subgroups have divergent transcriptional profiles, somatic mutations, copy number aberrations, and clinical outcomes (Morrissy *et al*., 2016; Northcott *et al*., 2012; Ramaswamy *et al*., 2013, 2016a,b). WNT and SHH MBs are clearly separable and identifiable across the majority of studies based on transcriptional and DNA methylation profiling data, demonstrating minimal overlap with other MB subgroups (Taylor *et al*., 2012). Group 3 and 4 MBs share several copy number alterations such as enrichment of isochromosome 17q, and the transcriptomes of group 3 and group 4 MBs are more similar to each other (Ramaswamy *et al*., 2016a,b; Taylor *et al*., 2012). The 2016 WHO classification of CNS tumors includes group 3 and group 4 MBs as provisional variants under the umbrellas of non‐WNT/non‐SHH MB (Louis *et al*., 2016). The survival rate of patients with MB largely depends on the molecular and clinical features of the cancer, varying from > 90% 5‐year overall survival (OS) for WNT MB patients to < 50% 5‐year OS for patients with metastatic group 3 or SHH MB with a TP53 mutation (Ramaswamy *et al*., 2016a,b). Aggressive yet nonspecific multimodal therapies (surgery, radiation therapy, and chemotherapy) have significantly improved the survival of MB patients, but survivors experience severe late‐onset cognitive and neurological side effects, including secondary malignancies (Crawford *et al*., 2007; Diller *et al*., 2009; Packer and Vezina, 2008; Packer *et al*., 2013). The cause of most MB‐related deaths is leptomeningeal metastases (Ramaswamy and Taylor, 2017). The relapse of MB is an almost uniformly fatal event, with no significant salvage rate (Ramaswamy and Taylor, 2017). It is essential to define the mechanisms of MB growth, metastasis, and recurrence to develop tailored therapies to selectively eradicate tumor cells responsible for MB expansion, metastasis, and relapse while sparing the developing brain (Vanner *et al*., 2014).

Stemness is defined as the potential for self‐renewal and differentiation from the cell of origin and was originally attributed to normal stem cells that have the ability to give rise to all cell types in the adult organism (Malta *et al*., 2018). Cancer stem cells (CSCs) are cancer cells that possess characteristics related to normal stem cells, specifically the ability to give rise to all tumor cell types (Bjerkvig *et al*., 2005). CSCs are considered to be responsible for tumor growth and maintenance, are often resistant to conventional chemotherapy and radiation therapy, and are involved in tumor metastasis and recurrence. Tumors are composed of a diverse, complex, integrated ecosystem of relatively differentiated tumor cells, CSCs, infiltrating immune cells, tumor‐associated fibroblasts, and endothelial cells, among other cell types (Malta *et al*., 2018). The tumor immune environment plays an important role in prognosis and response to therapy in various cancer types (Thorsson *et al*., 2018). MBs are cancers in which the majority of cells possess an undifferentiated stem‐ or progenitor‐like appearance (Fan and Eberhart, 2008), and CSCs have been identified from MB (Read *et al*., 2009; Singh *et al*., 2004; Ward *et al*., 2009). However, an integrated understanding of MB stemness, including the interface between the tumor immune environment and MB stemness, is lacking.

In this study, we analyzed cancer stemness in a cohort of primary MB samples (*n* = 763). First, we applied a trained stemness index model based on the previously existing one‐class logistic regression (OCLR) machine‐learning method (Malta *et al*., 2018; Sokolov *et al*., 2016), which includes a mRNA expression‐based signature and a DNA methylation‐based signature, to quantify MB stemness to acquire two independent stemness indices. One index [gene expression‐based stemness index (mRNAsi)] was reflective of gene expression; the other index (mDNAsi) was reflective of epigenetic features. Second, we assessed correlations between the two stemness indices and clinical and molecular features and identified a stemness molecular signature that might be helpful in guiding the prognostic status of MB patients. In addition, by applying CIBERSORT (Gentles *et al*., 2015) to profile immune cell types in MB, we gained insight into the interaction of the immune system with cancer stemness. Finally, using the Connectivity Map (CMap) (Subramanian *et al*., 2017), we discovered candidate compounds targeting the MB stemness signature.

## 2 Materials and methods

### 2.1 Data collection and processing

In this study, we collected 763 primary MB samples, which all had genome‐wide methylation and expression array data deposited in Gene Expression Omnibus (GEO) under the accession number GSE85218 (Cavalli *et al*., 2017), to analyze MB stemness. Microarray data from GSE85218 dataset were downloaded from GEO (http://www.ncbi.nlm.nih.gov/geo/query/acc.cgi?acc=GSE85218). Demographic and clinical information for the GSE85218 dataset is summarized in Table S1. For gene expression array data, background correction was carried out using the ‘backgroundCorrect’ function of the R package ‘limma’ with default parameters (Ritchie *et al*., 2015), and normalization was implemented with the ‘normalizeBetweenArrays’ function of the R package ‘limma’ with default parameters (Ritchie *et al*., 2015). The log2‐transformed normalized values of gene expression data were used to generate the mRNAsi. The DNA methylation level was represented using β values ranging from zero (no DNA methylation) to one (complete DNA methylation). For DNA methylation data, we excluded probes located on the sex chromosome and probes containing known single nucleotide polymorphisms. We performed normalization utilizing the SWAN method as part of the R package ‘minfi’ with default parameters (Maksimovic *et al*., 2012). β values were used to generate the mDNAsi.

### 2.2 Calculation of gene expression‐ and DNA methylation‐based stemness indices for MB

To calculate the mRNAsi and the DNA methylation‐based stemness index (mDNAsi), Malta *et al*. (2018) built a predictive model using an OCLR algorithm on pluripotent stem cell samples from the Progenitor Cell Biology Consortium dataset (Daily *et al*., 2017; Salomonis *et al*., 2016) to train two stemness signatures. The mRNA expression‐based signature contains a gene expression profile comprising 11 774 genes, and the DNA methylation‐based signature contains a set of 151 differentially methylated CpG sites. The work flow to generate the stemness indices (mRNAsi and mDNAsi) is available on https://bioinformaticsfmrp.github.io/PanCanStem_Web/. We applied the stemness index model to score the 763 MB samples using the same Spearman correlation (RNA expression data) or linear model (DNA methylation data) operators. The stemness indices were subsequently mapped to the [0,1] range via utilizing a linear transformation that subtracted the minimum and divided by the maximum. The MB samples stratified by the stemness indices were utilized for the integrative analyses.

### 2.3 Evaluation of associations between stemness indices and clinical outcomes in MB

We regarded the stemness index (mRNAsi or mDNAsi) as a single continuous covariate. The associations between the two stemness indices and OS in MB were assessed in three phases. First, we applied univariate Cox proportional hazard regression to calculate hazard ratios (HRs) for OS. The variables we included were mRNAsi, mDNAsi, age, sex, subgroup, tumor histology, metastatic status, and immune score. Immune score was calculated from gene expression data using the ESTIMATE algorithm (Yoshihara *et al*., 2013) and represents the level of infiltrating immune cells in any given MB sample. We found that only mRNAsi significantly correlated with OS for all MB patients. Therefore, mRNAsi was retained for subsequent analyses. Second, each subgroup of patients was split into low‐ and high‐risk groups based on the optimal cutoff value for mRNAsi, which was determined by using the ‘cutp’ function of the R package ‘survMisc’ (https://cran.r-project.org/web/packages/survMisc) with default parameters, and the survival difference between patients with high mRNAsi and low mRNAsi was compared by Kaplan–Meier (K‐M) survival plots. Finally, the statistically significant survival difference between patients with high mRNAsi and low mRNAsi was limited to SHH subgroup patients. We split the SHH MB dataset randomly into a 70% training set and 30% validation set splits by using the ‘createDataPartition’ function of the R package ‘caret’ (https://cran.r-project.org/web/packages/caret). The following nondefault parameters for the ‘createDataPartition’ function were used: *P* = 0.7 and list=FALSE. Distribution of clinical characteristics between the training and validation sets was compared with the Kruskal–Wallis test for continuous parameters and the chi‐square test for categorical parameters. In the training set, the differentially expressed genes (DEGs) between SHH subgroup samples with high mRNAsi and low mRNAsi were computed using the ‘lmFit’ function of the R package ‘limma’ with default parameters (Ritchie *et al*., 2015). In total, 3800 DEGs with an adjusted *P* value of < 0.05 were considered for the univariate Cox regression. The adjusted *P* value for multiple testing was computed utilizing the Benjamini–Hochberg (BH) correction. The univariate Cox regression analyses were performed to investigate the association between the OS of SHH MB patients in the training set and the expression level of each DEG. By performing the univariate Cox regression, 83 genes whose parameter *P* values were less than 0.001 were selected for subsequent analyses. In the training set, we employed Lasso‐penalized Cox regression analysis (Tibshirani, 1997) to further reduce genes for SHH MB patients. For the Lasso‐penalized Cox regression analysis, we subsampled the training set at a ratio of 7 : 3 with 1000 replacements and selected the genes with repeat occurrence frequencies of more than 200. A 23‐mRNA‐based risk score staging model was built based on a linear combination of the regression coefficient derived from the multivariate Cox regression (coef_*i*_) multiplied by its expression level (expr_*i*_). The formula for calculating risk scores is described as follows:

$$\text{Risk score}=\sum_{i=1}^{n}\left({\text{coef}}_{i}\times {\text{expr}}_{i}\right)$$

Sonic hedgehog subgroup samples were split into low‐ and high‐risk score groups according to the optimal cutoff value generated by using the ‘cutp’ function of the R package ‘survMisc’ with default parameters, and the two patient cohorts were compared by K‐M curves. To determine whether the predictive power of the 23‐mRNA‐based prognostic model could be independent of other clinical variables (including age, gender, histology, and metastatic status) for SHH MB patients, the multivariate Cox regression analyses were conducted. In the validation set, we applied the same risk score formula and cutoff point and divided the SHH MB patients into low‐ and high‐risk groups to test the robustness of the 23‐mRNA‐based prognostic model. We also employed the 23‐mRNA‐based prognostic model to predict survival of patients with other MB subgroups. Furthermore, we used a random model that was built by a random subset of 23 genes using the multivariate Cox regression analysis to predict survival of patients with SHH MB, and constructing the random model in the training set was also repeated 1000 times. The area under the curve (AUC) of the time‐dependent receiver operating characteristic (ROC) analysis was used to evaluate the predictive accuracy of the models.

### 2.4 Evaluation of associations between stemness indices and prognostic copy number alterations in SHH MB

The prognostic copy number alterations in SHH MB, including MYCN amplifications, GLI2 amplifications, and PTEN deletions, were calculated from DNA methylation arrays utilizing the R package ‘conumee’ with default parameters (http://bioconductor.org/packages/conumee) (Capper *et al*., 2018). *P* values for the associations between stemness indices and the prognostic copy number alterations of SHH MB were computed using Pearson's correlation coefficient tests followed by multiple testing using the BH method.

### 2.5 Evaluation of relationships between stemness indices and the MB immune microenvironment

By using CIBERSORT (a gene expression‐based deconvolution algorithm) (http://cibersort.stanford.edu/) (Gentles *et al*., 2015), we scored 22 immune cell types for their relative abundance in the MB samples. For any given MB sample, we computed the associations between mRNAsi/mDNAsi and the estimated fractions of individual immune cell types. By applying ESTIMATE (Yoshihara *et al*., 2013), we calculated the individual immune score to predict the level of infiltrating immune cells in any given MB sample. We calculated the correlations between mDNAsi/mRNAsi and immune score or PD‐L1 expression.

### 2.6 Identification of potential compounds targeting the MB stemness signature

We employed the recently updated CMap (September 2017) (Subramanian *et al*., 2017), a data‐driven, systematic approach for discovering correlations among genes, chemicals, and biological conditions, to search for candidate compounds that might target pathways correlated with MB stemness. In the CMap database, a total of 42 080 perturbagens were profiled to generate 473 647 reference signatures. The CMap workflow involves interrogating the CMap dataset of reference signatures with a query (a list of DEGs related to a biological state of interest) utilizing the pattern‐matching algorithms. The query results are scored ranging from −100 to 100. The molecule compounds are ranked according to their scores to yield most similar and opposing compounds. The CMap data and tools are available on https://clue.io. The DEGs between SHH subgroup samples with high mRNAsi and low mRNAsi were calculated using the ‘lmFit’ function of the R package ‘limma’ with default parameters (Ritchie *et al*., 2015). A list of genes differentially expressed between SHH subgroup samples with high mRNAsi and low mRNAsi was obtained, and the top 300 genes (150 upregulated and 150 downregulated) were selected to query the CMap database. Compounds with an enrichment score ≤ −95 were recorded as potential therapeutic agents for SHH MB.

### 2.7 Statistical analysis

r software version 3.4.4 (R Core Team, R Foundation for Statistical Computing, Vienna, Austria) was used for all statistical analyses. The OCLR method was implemented with the R package ‘gelnet’ with default parameters (Sokolov *et al*., 2016). *P* values for the associations between stemness indices and the MB immune microenvironment were computed using Pearson's correlation coefficient tests followed by multiple testing using the BH method. *P *< 0.05 was considered statistically significant.

## 3 Results

### 3.1 mRNA expression‐ and DNA methylation‐based stemness indices in MB

**Figure 1:**
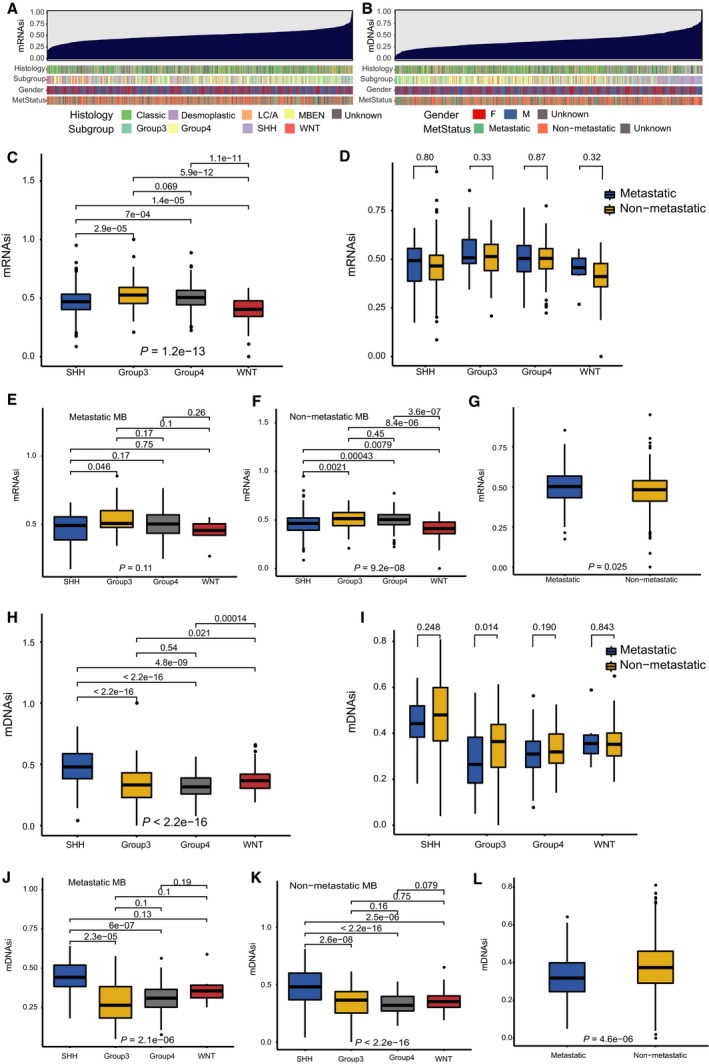
Clinical and molecular features associated with the mRNA expression‐based stemness index (mRNAsi) and the mDNAsi in MB. (A) An overview of the association between known clinical and molecular features (histology, subgroup, gender, and metastatic status) and mRNAsi in MB. Columns represent samples sorted by mRNAsi from low to high (top row). Rows represent known clinical and molecular features. (B) An overview of the association between known clinical and molecular features (histology, subgroup, gender, and metastatic status) and mDNAsi in MB. Columns represent samples sorted by mDNAsi from low to high (top row). Rows represent known clinical and molecular features. (C) Boxplots of mRNAsi in individual samples stratified by subgroup. (D) Boxplots of mRNAsi in individual samples from each MB subgroup, stratified by metastatic status. (E) Boxplots of mRNAsi in individual samples of patients with metastatic MB, stratified by subgroup. (F) Boxplots of mRNAsi in individual samples of patients with nonmetastatic MB, stratified by subgroup. (G) Boxplots of mRNAsi in individual samples stratified by metastatic status. (H) Boxplots of mDNAsi in individual samples stratified by subgroup. (I) Boxplots of mDNAsi in individual samples from each MB subgroup, stratified by metastatic status. (J) Boxplots of mDNAsi in individual samples of patients with metastatic MB, stratified by subgroup. (K) Boxplots of mDNAsi in individual samples of patients with nonmetastatic MB, stratified by subgroup. (L) Boxplots of mDNAsi in individual samples stratified by metastatic status. L/CA, large cell/anaplastic; MBEN, medulloblastoma with extensive nodularity; F, female; M, male; MetStatus, metastatic status.

We ranked the MB samples according to their mRNAsi or mDNAsi values (from low to high stemness index) and tested whether any demographic/molecular/clinical feature was correlated with either a low or high stemness index (Fig. 1A,B). We observed an inverse trend between mRNAsi and mDNAsi for subgroup and metastatic status (Fig. 1C–L). Group 3 and group 4 samples had higher mRNAsi values than WNT and SHH samples (Fig. 1C), while WNT and SHH samples had higher mDNAsi values than group 3 and group 4 samples (Fig. 1H). Similarly, patients with metastatic MB had higher mRNAsi values than patients with nonmetastatic MB (*P *=* *0.025, Fig. 1G), whereas the mDNAsi value was higher in patients with nonmetastatic MB than in patients with metastatic MB (*P *=* *4.6 × 10^−6^, Fig. 1L). In patients with group 3 MB, patients with nonmetastatic MB had higher mDNAsi values than patients with metastatic MB (*P *=* *0.014, Fig. 1I). In addition, in patients with nonmetastatic MB, the mRNAsi value was higher in patients with group 3 MB and patients with group 4 MB (Fig. 1F), while the mDNAsi was highest in patients with SHH MB (Fig. 1K). In patients with metastatic MB, the mDNAsi was highest in patients with SHH MB (Fig. 1J).

### 3.2 Correlations of stemness indices with clinical outcome in MB

**Figure 2:**
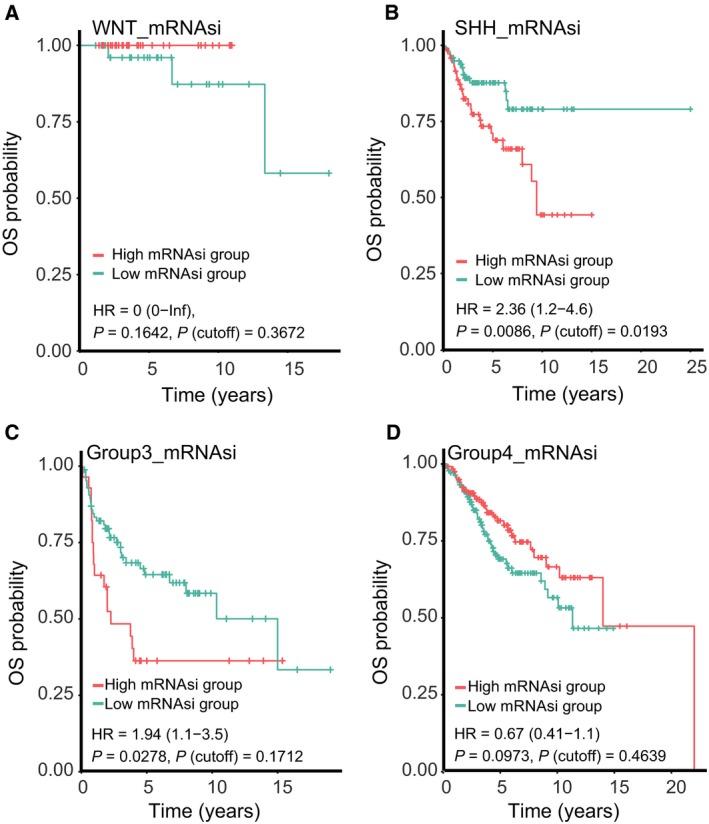
K‐M curves showing the OS of each subgroup of MB patients with high or low mRNAsi. The K‐M survival curves show the OS based on the high and low mRNAsi patients divided by the optimal cutoff point. (A) K‐M curve showing the OS of WNT MB patients with a high or low mRNAsi. (B) K‐M curve showing the OS of SHH MB patients with a high or low mRNAsi. (C) K‐M curve showing the OS of group 3 MB patients with a high or low mRNAsi. (D) K‐M curve showing the OS of group 4 MB patients with a high or low mRNAsi.

**Figure 3:**
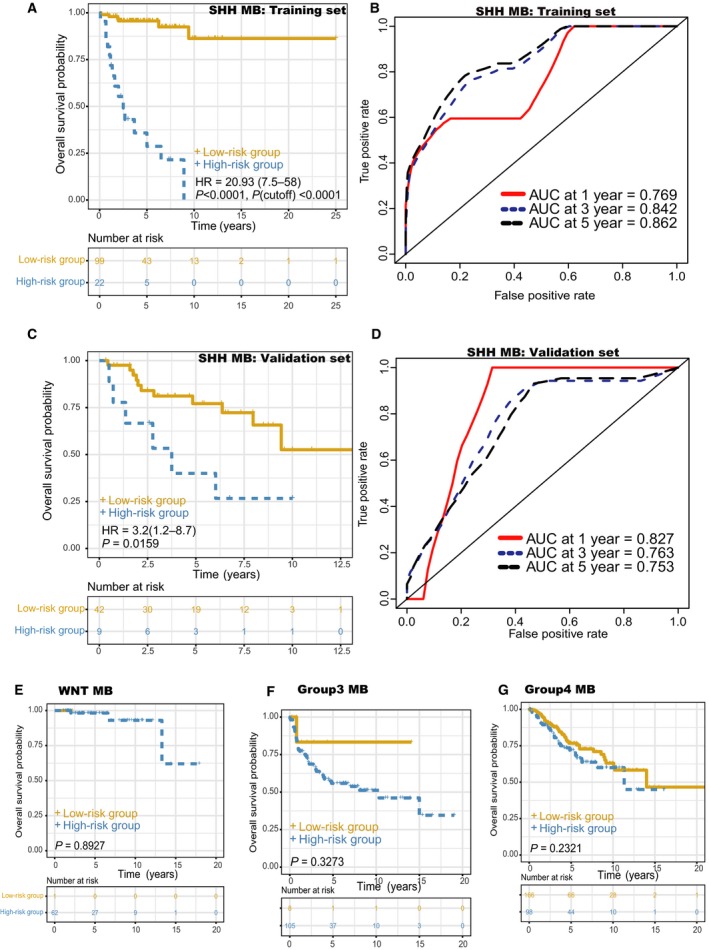
Prognostic value of the 23‐mRNA‐based prognostic model in patients stratified by MB subgroup. The K‐M survival curves show the OS based on the high‐ and low‐risk groups divided by the optimal cutoff point. (A) K‐M curves for the training set of SHH MB patients. (B) Time‐dependent ROC curves showed the predictive efficiency of the 23‐mRNA‐based prognostic model in the training set of SHH MB patients. (C) K‐M curves for the validation set of SHH MB patients. (D) Time‐dependent ROC curves showed the predictive efficacy of the 23‐mRNA‐based prognostic model in the validation set of SHH MB patients. (E) K‐M curves for the WNT MB patients. (F) K‐M curves for the group 3 MB patients. (G) K‐M curves for the group 4 MB patients.

**Figure 4:**
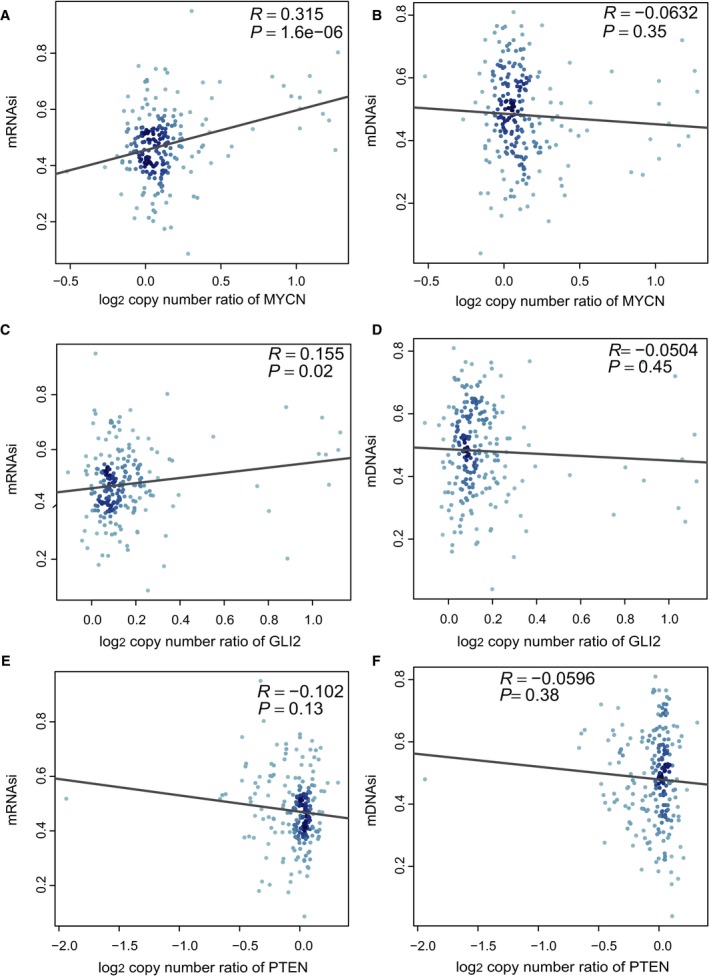
Associations of stemness indices with the prognostic copy number alterations in SHH MB. (A) Correlation between mRNAsi and MYCN amplification. (B) Correlation between mDNAsi and MYCN amplification. (C) Correlation between mRNAsi and GLI2 amplification. (D) Correlation between mDNAsi and GLI2 amplification. (E) Correlation between mRNAsi and PTEN deletion. (F) Correlation between mDNAsi and PTEN deletion.

By using the univariable Cox regression analyses, we found that mRNAsi had a statistically significant effect on OS for MB (HR, 11.43; 95% CI, 2.79–46.76; *P *=* *7.03 × 10^−4^), whereas mDNAsi had no significant association with OS for MB (Table 1). For each subgroup of MB patients, the statistically significant OS difference between patients with high mRNAsi and low mRNAsi was restricted to SHH MB patients [HR, 2.36; 95% CI, 1.2–4.6; *P *=* *0.0086; *P* (cutoff) = 0.0193] (Fig. 2). Distribution of clinical characteristics [age (*P* = 0.24), gender (*P* = 0.66), histology (*P* = 0.21), metastatic status (*P* = 0.83)] was balanced between the training and validation sets (Table S2). The genes differentially expressed between SHH MB samples with high mRNAsi and low mRNAsi were screened, and univariate, Lasso, and multivariate Cox analyses were conducted to construct a 23‐mRNA‐based prognostic model. The gene expression‐based prognostic model was characterized by the linear combination of the expression values of the 23 genes weighted by their relative coefficients in the multivariate Cox regression analysis. Table S3 shows the multivariate Cox regression coefficients of the genes in the 23‐mRNA‐based prognostic model. In this 23‐mRNA‐based prognostic model, higher expression levels of ADAMTSL3, CPE, EFEMP2, FAM214A, FKBP4, FRZB, HIST1H2APS4, ITIH2, KCNG1, LPCAT3, MTRR, NLGN4Y, and TIMM50 were related to a lower risk of death (coefficient < 0). In contrast, higher expression levels of COLGALT1, KIAA0825, LDB3, PIP4K2A, PROSER1, TMEM185B, TMEM38B, TOMM40, TRIM28, and TRMT1 were associated with worse OS (coefficient > 0). By applying this prognostic model, each patient with SHH MB was given a risk score in connection with individual prognosis. Then, patients with SHH MB in the training set were classified into a high‐risk group (*n* = 22) and a low‐risk group (*n* = 99) by the cutoff value for the 23‐mRNA‐based risk scores. The K‐M OS curves of the two groups in the training set, based on the 23 genes, were significantly different (HR, 20.93; 95% CI, 7.5–58; *P *<* *0.0001; Fig. 3A). The predictive capacity of the 23‐mRNA‐based prognostic model was assessed by calculating the AUC of an ROC curve. The AUCs of the 23‐gene biomarker prognostic model in the training set were 0.769, 0.842, and 0.862 for the 1‐, 3‐, and 5‐year survival times, respectively (Fig. 3B). We incorporated age, gender, histology, metastatic status, and the 23‐mRNA‐based prognostic model into the multivariate Cox regression analysis. Based on the multivariate Cox regression analysis, the 23‐mRNA‐based prognostic model was an independent prognostic factor correlated with OS (Table 2). Ultimately, in the validation set, patients with SHH MB were classified into a high‐risk group (*n* = 9) and a low‐risk group (*n* = 42). The K‐M OS curves of the two groups in the validation set were significantly different (HR, 3.2; 95% CI, 1.2–8.7; *P *=* *0.0159; Fig. 3C). The AUCs of the 23‐gene biomarker prognostic model in the validation set were 0.827, 0.763, and 0.753 for the 1‐, 3‐, and 5‐year survival times, respectively (Fig. 3D). When we applied the 23‐mRNA‐based prognostic model to predict the survival of patients with other MB subgroups, we found that there were no statistically significant differences in OS between the high‐risk group and the low‐risk group (Fig. 3E–G), indicating that the 23‐mRNA‐based signature is not applicable to other MB subgroups. The 23‐mRNA‐based signature had a much higher predictive accuracy than a random model based on a random subset of 23 genes (Table S4). Moreover, we found the positive correlations between mRNAsi and the prognostic copy number alterations in SHH MB, including MYCN amplifications and GLI2 amplifications (Fig. 4A,C). However, we found no significant correlations between mDNAsi and the prognostic copy number alterations in SHH MB, including MYCN amplifications (Fig. 4B), GLI2 amplifications (Fig. 4D), and PTEN deletions (Fig. 4F), and there was no statistically significant association between mRNAsi and PTEN deletions in SHH MB (Fig. 4E).

**Table 1:** Univariate Cox regression analyses of clinical and molecular features associated with OS of MB patients. LC/A, large cell/anaplastic; MBEN, medulloblastoma with extensive odularity.

Variable	HR (95% CI)	*P*
mRNAsi		
Increasing mRNAsi	11.43 (2.79–46.76)	**7.03E‐04**
mDNAsi		
Increasing mDNAsi	0.65 (0.20–2.07)	0.468
Immune score		
Increasing immune scores	1.0000 (0.9996–1.0005)	0.822
Age		
Increasing years	0.98 (0.96–1.00)	0.092
Gender		
Male vs female	1.17 (0.83–1.63)	0.368
Metastatic status		
Metastatic vs nonmetastatic	1.65 (1.18–2.30)	**0.004**
Subgroup		
SHH vs WNT	5.26 (1.62–17.05)	**0.006**
Group 3 vs WNT	10.87 (3.38–34.92)	**6.2E‐05**
Group 4 vs WNT	6.02 (1.90–19.11)	**0.002**
Histology		
LC/A vs MBEN	4.23 (1.01–17.82)	**0.049**
Desmoplastic vs MBEN	1.06 (0.24–4.74)	0.939
Classic vs MBEN	1.77 (0.43–7.19)	0.426

**Table 2:** Multivariate Cox regression analysis of the 23‐mRNA‐based prognostic model and clinical features associated with OS of SHH MB patients. LC/A, large cell/anaplastic; MBEN, medulloblastoma with extensive nodularity

Variables	HR (95% CI)	*P*
Age		
Increasing years	1.00 (0.97–1.04)	0.858
Gender		
Male vs female	0.51 (0.23–1.12)	0.095
Histology		
Desmoplastic vs classic	0.31 (0.10–0.98)	**0.046**
LC/A vs classic	0.45 (0.16–1.25)	0.123
MBEN vs classic	0.00 (0–Inf)	0.997
Metastatic status		
Metastatic vs nonmetastatic	3.40 (1.36–8.48)	**0.009**
23‐mRNA‐based prognostic model		
Increasing risk scores	1.80 (1.45–2.24)	**1.10E‐07**

### 3.3 Association of the stemness indices with the MB immune microenvironment

**Figure 5:**
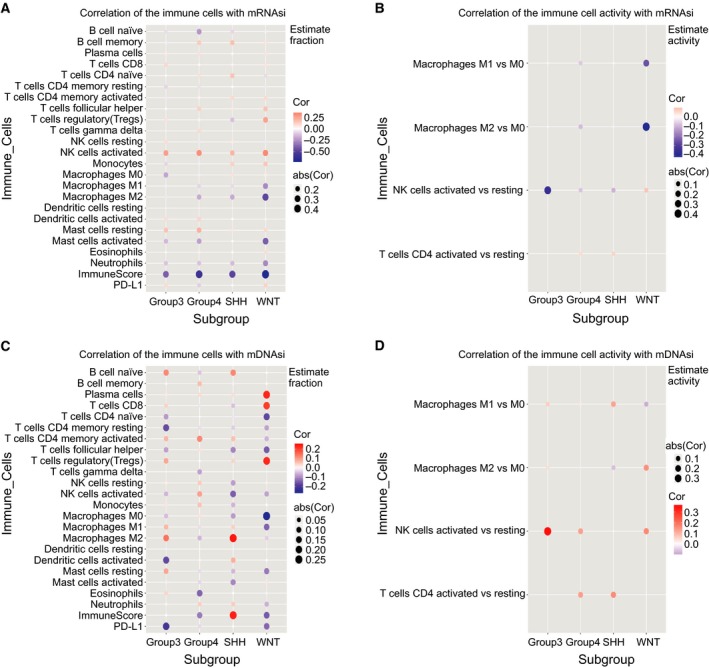
Associations of stemness indices with the immune microenvironment in each subgroup of MB. (A) Plots show correlations between the mRNAsi and CIBERSORT estimates of immune cell subpopulation fractions and PD‐L1 protein expression. (B) Plots show correlations between the mRNAsi and estimated immune cell activity, computed as the difference between the fractions of activated and resting populations. The correlations are included for macrophages, NK cells, and CD4^+^ T cells. (C) Plots show correlations between the mDNAsi and CIBERSORT estimates of immune cell subpopulation fractions and PD‐L1 protein expression. (D) Similar to (B), plots show correlations between mDNAsi and estimated immune cell activity.

To assess the relationships between MB stemness and the tumor microenvironment, we computed correlations between individual types of immune cells and mRNAsi (Fig. 5A,B and Table 3) and mDNAsi (Fig. 5C,D and Table 4). For WNT subgroup MBs (Fig. 5A and Table 3), the mRNAsi was correlated positively with the fraction of activated natural killer (NK) cells [*R* = 0.318, *P* (adjusted) = 0.036] and negatively with the fractions of M2 macrophages [*R* = −0.468, *P* (adjusted) = 0.001] and activated mast cells [*R* = −0.396, *P* (adjusted) = 0.004]. For the SHH subgroup MBs (Fig. 5A and Table 3), mRNAsi was positively associated with the fraction of activated NK cells [*R* = 0.192, *P* (adjusted) = 0.0496] and negatively associated with the fraction of M2 macrophages [*R* = −0.184, *P* (adjusted) = 0.0496]. Group 3 subgroup MBs exhibited a positive correlation between mRNAsi and the fraction of activated NK cells [*R* = 0.257, *P* (adjusted) = 0.024] (Fig. 5A and Table 3). For group 4 MBs (Fig. 5A and Table 3), the mRNAsi was related positively to the fractions of activated NK cells [*R* = 0.300, *P* (adjusted) = 4.52 × 10^−7^) and resting mast cells [*R* = 0.202, *P* (adjusted) = 0.001] and was related negatively to the fractions of naïve B cells [*R* = −0.237, *P* (adjusted) = 0.0001], M2 macrophages [*R* = −0.184, *P* (adjusted) = 0.004], and activated mast cells [*R* = −0.213, *P* (adjusted) = 0.001]. Except for the positive association between mDNAsi and M2 macrophages for SHH subgroup MBs (*R* = 0.248, *P* (adjusted) = 0.005) (Fig. 5C and Table 4), there were no significant associations between mDNAsi and the fractions of 22 immune cell types in any of the four subgroups. In addition, we found no significant correlations between the stemness indices and PD‐L1 expression in any of the subgroups (Fig. 5A,C and Table 3, 4). The mRNAsi (Fig. 5A and Table 3) had an inverse correlation with immune score for the WNT subgroup [*R* = −0.695, *P* (adjusted) = 6.43 × 10^−10^], SHH subgroup [*R* = −0.474, *P* (adjusted) = 1.89 × 10^−12^], group 3 subgroup [*R* = −0.398, *P* (adjusted) = 1.99 × 10^−5^], and group 4 subgroup [*R* = −0.587, *P* (adjusted) = 3.70 × 10^−30^], and mDNAsi (Fig. 5C and Table 4) had only a positive correlation with immune score for the SHH subgroup [*R* = 0.236, *P* (adjusted) = 0.005].

**Table 3:** Correlations of mRNAsi with immune microenvironment in each subgroup MB.

Immune cell	WNT			SHH			Group 3			Group 4		
	*r*	*P*	*P* a	*r*	*P*	*P* a	*r*	*P*	*P* a	*r*	*P*	*P* a
B cells (naive)	−0.030	0.807	0.840	−0.083	0.214	0.557	−0.068	0.420	0.709	−0.237	0.000	**0.0001**
B cells (memory)	0.063	0.605	0.796	0.162	0.015	0.100	−0.031	0.711	0.912	0.135	0.014	0.056
Plasma cells	0.066	0.586	0.796	0.015	0.821	0.928	0.046	0.584	0.872	0.072	0.195	0.330
T cells (CD8)	0.064	0.600	0.796	−0.021	0.751	0.928	0.100	0.231	0.691	0.012	0.828	0.828
T cells (CD4 naive)	−0.070	0.567	0.796	0.154	0.022	0.113	0.003	0.971	0.993	0.075	0.179	0.322
T cells [CD4 memory (resting)]	−0.042	0.728	0.834	0.006	0.932	0.932	−0.086	0.306	0.691	−0.060	0.278	0.416
T cells [CD4 memory (activated)]	0.089	0.464	0.796	0.091	0.177	0.511	−0.042	0.620	0.872	0.025	0.657	0.705
T cells (follicular helper)	0.158	0.191	0.478	0.051	0.450	0.782	0.003	0.967	0.993	0.117	0.035	0.104
T cells (regulatory (Tregs))	0.245	0.041	0.113	−0.131	0.051	0.188	0.067	0.425	0.709	0.026	0.634	0.705
T cells (gamma delta)										0.089	0.109	0.245
NK cells (resting)	−0.041	0.734	0.834	0.046	0.493	0.801	0.082	0.329	0.691	−0.045	0.417	0.563
NK cells (activated)	0.318	0.007	**0.036**	0.192	0.004	**0.0496**	0.257	0.002	**0.024**	0.300	0.000	**4.52E**−**07**
Monocytes	0.145	0.232	0.527	0.117	0.082	0.265	−0.084	0.320	0.691	−0.033	0.556	0.683
Macrophages (M0)	0.041	0.733	0.834	0.069	0.304	0.659	−0.200	0.016	0.137	−0.030	0.584	0.686
Macrophages (M1)	−0.275	0.021	0.076	−0.072	0.282	0.659	−0.029	0.729	0.912	−0.087	0.119	0.246
Macrophages (M2)	−0.468	0.000	**0.001**	−0.184	0.006	**0.0496**	−0.041	0.627	0.872	−0.184	0.001	**0.004**
Dendritic cells (resting)												
Dendritic cells (activated)	0.034	0.779	0.840	0.009	0.892	0.928	0.081	0.332	0.691	0.105	0.058	0.155
Mast cells (resting)	0.090	0.460	0.796	0.056	0.401	0.782	0.155	0.064	0.361	0.202	0.000	**0.001**
Mast cells (activated)	−0.396	0.001	**0.004**	0.011	0.876	0.928	−0.150	0.072	0.361	−0.213	0.000	**0.001**
Eosinophils	0.081	0.504	0.796	−0.009	0.891	0.928	0.001	0.993	0.993	0.035	0.531	0.683
Neutrophils	−0.277	0.020	0.076	−0.142	0.034	0.149	−0.122	0.144	0.600	−0.127	0.022	0.075
Immune score	−0.695	0.000	**6.43E‐10**	−0.474	0.000	**1.89E‐12**	−0.398	0.000	**1.99E‐05**	−0.587	0.000	**3.70E‐30**
PD‐L1	0.109	0.369	0.768	−0.015	0.824	0.928	0.084	0.315	0.691	−0.098	0.078	0.190
Macrophages (M1 vs M0)	−0.255	0.039	0.113	0.013	0.882	0.928	0.014	0.866	0.993	−0.067	0.256	0.407
Macrophages (M2 vs M0)	−0.407	0.001	**0.004**	−0.016	0.862	0.928	−0.007	0.932	0.993	−0.084	0.156	0.300
NK cells (activated vs resting)	0.087	0.870	0.870	−0.095	0.630	0.928	−0.362	0.425	0.709	−0.073	0.679	0.705
T cells (CD4 activated vs resting)				0.062	0.451	0.782				0.050	0.414	0.563

^a^A false discovery rate (FDR) correction using the BH method is applied to *P* values.

**Table 4:** Correlations of mDNAsi with immune microenvironment in each subgroup MB.

Immune cell	WNT subgroup			SHH subgroup			Group 3 subgroup			Group 4 subgroup		
	*r*	*P*	*P* a	*r*	*P*	*P* a	*r*	*P*	*P* a	*r*	*P*	*P* a
B cells (naïve)	−0.002	0.989	0.989	0.121	0.071	0.372	0.118	0.159	0.796	−0.044	0.424	0.828
B cells (memory)	0.005	0.968	0.989	−0.010	0.877	0.981	−0.004	0.961	0.974	0.063	0.256	0.828
Plasma cells	0.231	0.055	0.484	0.030	0.652	0.808	0.019	0.824	0.974	0.031	0.581	0.828
T cells (CD8)	0.205	0.089	0.557	−0.046	0.492	0.723	0.040	0.630	0.893	−0.017	0.758	0.861
T cells (CD4 naive)	−0.172	0.154	0.637	−0.018	0.788	0.931	−0.084	0.318	0.880	0.001	0.992	0.992
T cells (CD4 memory (resting))	−0.077	0.526	0.949	−0.037	0.583	0.758	−0.167	0.045	0.376	−0.024	0.664	0.828
T cells (CD4 memory (activated))	−0.061	0.617	0.964	0.061	0.362	0.723	0.071	0.398	0.880	0.119	0.032	0.236
T cells (follicular helper)	−0.151	0.211	0.637	−0.106	0.116	0.439	−0.078	0.356	0.880	0.024	0.665	0.828
T cells (regulatory (Tregs))	0.228	0.058	0.484	0.041	0.539	0.738	0.085	0.313	0.880	0.002	0.968	0.992
T cells (gamma delta)										−0.075	0.177	0.797
NK cells (resting)	0.015	0.900	0.989	−0.081	0.229	0.587	0.015	0.854	0.974	0.049	0.380	0.828
NK cells (activated)	−0.076	0.531	0.949	−0.147	0.029	0.247	−0.057	0.499	0.892	0.093	0.094	0.507
Monocytes	0.005	0.968	0.989	−0.058	0.385	0.723	0.023	0.782	0.974	0.058	0.293	0.828
Macrophages (M0)	−0.258	0.031	0.484	−0.083	0.216	0.587	−0.051	0.546	0.893	0.024	0.668	0.828
Macrophages (M1)	−0.140	0.246	0.637	0.045	0.501	0.723	0.069	0.414	0.880	−0.024	0.672	0.828
Macrophages (M2)	−0.037	0.764	0.989	0.248	0.000	**0.005**	0.142	0.090	0.563	−0.058	0.294	0.828
Dendritic cells (resting)												
Dendritic cells (activated)	0.003	0.981	0.989	0.078	0.248	0.587	−0.171	0.041	0.376	0.011	0.840	0.907
Mast cells (resting)	−0.118	0.331	0.752	−0.056	0.408	0.723	0.086	0.304	0.880	−0.025	0.648	0.828
Mast cells (activated)	0.022	0.856	0.989	−0.105	0.118	0.439	−0.011	0.899	0.974	−0.030	0.589	0.828
Eosinophils	−0.010	0.934	0.989	−0.002	0.981	0.981	0.039	0.643	0.893	−0.140	0.012	0.236
Neutrophils	−0.064	0.600	0.964	0.049	0.468	0.723	0.010	0.901	0.974	0.045	0.419	0.828
Immune score	−0.151	0.213	0.637	0.236	0.000	**0.005**	0.003	0.974	0.974	−0.117	0.035	0.236
PD‐L1	−0.138	0.255	0.637	0.003	0.960	0.981	−0.202	0.015	0.376	−0.027	0.629	0.828
Macrophages (M1 vs M0)	−0.089	0.479	0.949	0.123	0.175	0.569	0.064	0.457	0.880	0.025	0.675	0.828
Macrophages (M2 vs M0)	0.150	0.229	0.637	−0.069	0.449	0.723	0.040	0.639	0.893	−0.018	0.765	0.861
NK cells (activated vs resting)	0.160	0.762	0.989	0.005	0.978	0.981	0.355	0.435	0.880	0.129	0.461	0.828
T cells (CD4 activated vs resting)				0.157	0.054	0.351				0.131	0.030	0.236

^a^A false discovery rate (FDR) correction using the BH method is applied to *P* values.

### 3.4 Connectivity Map analysis identifies novel candidate compounds targeting the MB stemness signature

**Figure 6:**
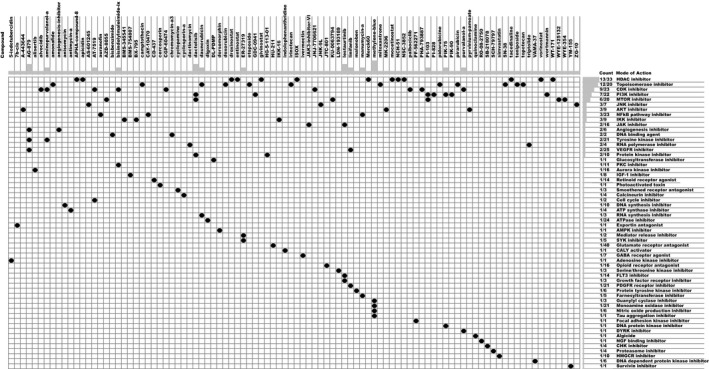
Heatmap showing each compound (perturbagen) from the CMap that shares a MoA (rows), sorted by descending number of compounds with a shared MoA. The above compounds have an enrichment score ≤ −95 and might be capable of targeting the MB stemness signature.

To identify potential compounds capable of targeting the pathways associated with MB stemness, we queried the CMap database using the mRNA expression signatures by applying differential expression analysis to SHH subgroup samples with high mRNAsi and low mRNAsi values. The top 96 compounds that were able to repress the above gene expression profile of SHH MB are shown in Fig. 6 and Table S5. CMap mode of action (MoA) analysis of the 96 compounds revealed 58 mechanisms of action shared by the above compounds. Thirteen compounds (APHA‐compound‐8, apicidin, droxinostat, entinostat, givinostat, ISOX, Merck60, mocetinostat, NCH‐51, NSC‐3852, tacedinaline, vorinostat, and WT‐171) shared the MoA of HDAC inhibitors, and 12 compounds (amonafide, camptothecin, daunorubicin, doxorubicin, etoposide, irinotecan, mitoxantrone, pidorubicine, pirarubicin, SN‐38, teniposide, and topotecan) shared the MoA of topoisomerase inhibitors. We found that alvocidib, aminopurvalanol‐a, AT‐7519, bisindolylmaleimide‐ix, CGP‐60474, JNJ‐7706621, palbociclib, PHA‐793887, and purvalanol‐a shared the MoA of CDK inhibitors, and dactolisib, GDC‐0941, PI‐103, PI‐828, PIK‐75, PIK‐90, and wortmannin shared the MoA of PI3K inhibitors. Moreover, 6 compounds (AZD‐8055, dactolisib, KU‐0063794, PI‐103, WYE‐125132, and WYE‐354) shared the MoA of MTOR inhibitors.

## 4 Discussion

Leveraging a large cohort of primary MBs profiled based on combined DNA methylation and gene expression, we performed a comprehensive analysis of MB stemness. By employing a stemness index model‐based OCLR machine‐learning algorithm to the MB samples, we obtained two distinct molecular metrics of stemness and then applied these metrics to assess the epigenomic and transcriptomic stemness features of MBs based on their molecular and clinical information. Moreover, we identified a 23‐mRNA‐based prognostic model that could effectively predict the survival of SHH MB patients and revealed the positive correlations between mRNAsi and the prognostic copy number changes in SHH MB, including MYCN amplifications and GLI2 amplifications. Using CIBERSORT, we obtained insight into the interaction of MB stemness and the immune microenvironment. Taking advantage of CMap, we identified potential drugs targeting SHH MB stem cells. With regard to the association between stemness indices and prognosis in MB patients, we showed that mRNAsi had a positive correlation with MB subgroup and a significant association with OS, while mDNAsi had a negative correlation with MB subgroup and no significant association with OS, suggesting that mRNAsi could recapitulate prognostic molecular subgroups of MB. According to mRNAsi, only patients with SHH MB could be divided into two groups with distinct prognoses, indicating that the SHH subgroup might have a higher degree of intrasubgroup heterogeneity than other subgroups with respect to the stemness phenotype. Previous studies have shown that WNT, SHH, and group 4 MBs have different cellular origins (Gibson *et al*., 2010; Lin *et al*., 2016; Schüller *et al*., 2008; Yang *et al*., 2008). Given that the cancer methylome can reflect the cell of origin (Fernandez *et al*., 2012; Hovestadt *et al*., 2014), different mDNAsi of MB subgroups may provide additional evidence for distinct cellular origins for MB subgroups.

Stem cell signatures shared by leukemia and hematopoietic stem cells predict clinical outcomes in acute myeloid leukemia patients (Eppert *et al*., 2011). Similarly, in colon, breast, and non‐small‐cell lung cancer, stem cell signature expression correlates inversely with patient survival (Liu *et al*., 2007; Merlos‐Suárez *et al*., 2011; Zheng *et al*., 2013a,b). Moreover, a medulloblastoma‐propagating cell signature defines SHH MB patients with a poor prognosis (Vanner *et al*., 2014). These studies revealed that for multiple tumors, including MB, patients whose cancer exhibits higher expression levels of stem cell genes experience significantly worse clinical outcomes. In the present study, we built and validated a 23‐mRNA‐based prognostic model associated with stem cell genes. To our knowledge, all predictive genes in this 23‐mRNA signature have not been reported for MB and may provide some clinical indications for the development of novel prognostic factors for MB. One of the advantages of predictive genes is that they do not require the identification of somatic mutations in patients and reduce the cost of sequencing, which may make the application of panel testing based on specific mRNAs more routine. Additionally, when applied to single‐cell transcriptomic profiles of MB, the stemness indices could reveal intratumor heterogeneity for the stemness of individual MB cells and identify the MB cells that exhibit greater proliferation and tumor‐propagating potential.

We found that mRNAsi had a negative association with the immune score for all of the MB subgroups, suggesting that immune cells in MB may repress MB stem cells by affecting the transcriptome of MB stem cells. In addition, the excellent prognosis of WNT MB may be explained in part by the result that the negative correlation between mRNAsi and immune score was stronger in the WNT subgroup than in the other subgroups. The absence of PD‐L1 expression in MB (Aoki *et al*., 2016; Majzner *et al*., 2017; Vermeulen *et al*., 2018) might explain in part why the stemness indices had no significant associations with PD‐L1 expression, indicating that the therapeutic potential of immunotherapy with PD‐L1 inhibitors seems limited in MB. For all of the MB subgroups, the mRNAsi was associated positively with the fraction of activated NK cells, suggesting that NK cells may promote MB stem cell‐associated phenotypes and that the added value of NK cell‐based therapies in MB may be limited. We observed that the fractions of M2 macrophages in WNT, SHH, and group 4 MBs were negatively associated with mRNAsi, indicating that M2 macrophages might suppress MB stem cells by impacting the transcriptome of MB stem cells. A recent study showed that M1 rather than M2 macrophages correlate more strongly with worse clinical outcome in SHH MB (Lee *et al*., 2018). These two results contradict the common view of tumor‐promoting M2 macrophages and tumor‐suppressing M1 macrophages. In many cancer types, M2 macrophage counts are associated with adverse outcomes (Hu *et al*., 2016; Jensen *et al*., 2009; Kawachi *et al*., 2018; Medrek *et al*., 2012), and M1 macrophage infiltration is correlated with better prognosis (Ma *et al*., 2010; Mei *et al*., 2016). However, several studies suggest that the dichotomous M1/M2 classification of macrophages is oversimplified, and the role of tumor‐associated macrophages is still controversial (Martinez and Gordon, 2014; Van Overmeire *et al*., 2014). Furthermore, our analyses showed only weak associations between mDNAsi and immune cells in MB. This result suggests that immune cells in MB are likely to have a weak effect on the methylome of MB stem cells.

We interrogated CMap utilizing the gene expression signatures from SHH MB samples with high and low mRNAsi levels. The CMap analysis precisely identified some compounds that have been shown to specifically impact CSCs in other tumor types (Angeletti *et al*., 2016; Batsaikhan *et al*., 2014; Battula *et al*., 2017; Bonuccelli *et al*., 2017; Bozok Cetintas *et al*., 2016; Chen *et al*., 2015, 2016; Cheng *et al*., 2017; Dominguez‐Gomez *et al*., 2018; Garulli *et al*., 2014; Hong *et al*., 2011; Hou *et al*., 2018; Malkomes *et al*., 2016; Xiang *et al*., 2017; Xu *et al*., 2016; Yeh *et al*., 2013; Yin *et al*., 2018; You *et al*., 2009; Zhang *et al*., 2013; Zheng *et al*., 2013a,b). These compounds include the CDK inhibitors palbociclib and alvocidib, the AMPK inhibitor dorsomorphin, the IKK inhibitor BMS‐345541, the smoothened receptor antagonist cyclopamine, the topoisomerase inhibitors topotecan and doxorubicin, the GABA receptor agonist ivermectin, the NF‐κB pathway inhibitor auranofin, the MTOR inhibitor dactolisib, the AKT inhibitors MK‐2206 and pyrvinium‐pamoate, the HMGCR inhibitor simvastatin, the HDAC inhibitors apicidin, vorinostat, and givinostat, and the DNA synthesis inhibitor anisomycin. In addition, the survivin inhibitor YM155 (Brun *et al*., 2015), the AKT inhibitor pyrvinium (Li *et al*., 2014), and the RNA polymerase inhibitor triptolide (Zhang *et al*., 2018) have been shown to exert anticancer effects on MB cells, although there were no results regarding effects on MB stem cells. More importantly, the CMap analysis identified the PI3K inhibitor GDC‐0941, which has been demonstrated to target CD133‐positive stem cell‐like MB subpopulations (Ehrhardt *et al*., 2015). The mentioned compounds may present an avenue for the implementation of targeting MB stem cells. Given that the survival rates of MB patients treated with nonspecific multimodal therapies have reached a plateau (Ramaswamy and Taylor, 2017), targeting MB stem cells in parallel to nonspecific multimodal therapies may yield the most durable SHH MB remission.

However, several limitations should be acknowledged for the current study. First, the ethnicities of populations in the GSE85218 dataset are primarily limited to Caucasian and African American, and the extrapolation of our findings to other ethnic groups needs to be further substantiated. Second, the 23‐mRNA‐based signature was not subjected to external validation because the appropriate independent cohorts with survival data were not available, and a robust signature should be validated externally in different datasets; thus, the prospective multicenter clinical trials are required to further validate the findings. Finally, the mechanisms underlying our findings have not been clearly elucidated here, and experimental studies on our findings should be carried out to facilitate our understanding of their functional roles in MB and their clinical application.

## 5 Conclusions

Taken together, our results provide a comprehensive characterization of MB stemness. The prognostic signature based on mRNAsi may contribute to personalized prediction of SHH MB prognosis and act as a potential biomarker for SHH MB prognostication and response to differentiation therapies in clinical practice. Our study also provides strategies based on machine‐learning methods for the systematic identification of biomarkers that stratify MB in terms of MB stemness and drugs targeting MB stem cells. Our analysis regarding the interactions of tumor‐infiltrating immune cells with MB stemness may help predict the efficacy of immunotherapies targeting MB stem cells and contribute to the identification of patients who will respond to such therapies. Future investigations should concentrate on the functional explanation of our results and the validation of our findings in planned clinical trials.

## Conflict of interest

The authors declare no conflict of interest.

## Author contributions

HL drafted the manuscript. HL, YH, YZ, and SY prepared all figures and tables. All authors reviewed and approved the final manuscript.

## Data Accessibility

Source codes used for our data analysis are available at https://github.com/richie2019/MBpanel.

## Supporting information


**Table S1.** Clinicopathological features of patients in the GSE85218 dataset.
**Table S2.** Comparison of distribution of clinical characteristics between the training and validation set.
**Table S3.** The multivariate Cox regression coefficients of the genes in the 23‐mRNA‐based prognostic model.
**Table S4.** Comparisons of the predictive value of the 23‐mRNA‐based prognostic model and the random model based on a random subset of 23 genes.
**Table S5.** Compounds with an enrichment score ≤ −95 that could target pathways associated with MB stemness.

## References

[mol212557-bib-0001] Angeletti F , Fossati G , Pattarozzi A , Würth R , Solari A , Daga A , Masiello I , Barbieri F , Florio T and Comincini S (2016) Inhibition of the autophagy pathway synergistically potentiates the cytotoxic activity of givinostat (ITF2357) on human glioblastoma cancer stem cells. Front Mol Neurosci 9, 107. 27833530 10.3389/fnmol.2016.00107 PMC5081386

[mol212557-bib-0002] Aoki T , Hino M , Koh K , Kyushiki M , Kishimoto H , Arakawa Y , Hanada R , Kawashima H , Kurihara J , Shimojo N *et al* (2016) Low frequency of programmed death ligand 1 expression in pediatric cancers. Pediatr Blood Cancer 63, 1461–1464. 27135656 10.1002/pbc.26018 PMC5074238

[mol212557-bib-0003] Batsaikhan BE , Yoshikawa K , Kurita N , Iwata T , Takasu C , Kashihara H and Shimada M (2014) Cyclopamine decreased the expression of Sonic Hedgehog and its downstream genes in colon cancer stem cells. Anticancer Res 34, 6339–6344. 25368233

[mol212557-bib-0004] Battula VL , Nguyen K , Sun J , Pitner MK , Yuan B , Bartholomeusz C , Hail N and Andreeff M (2017) IKK inhibition by BMS‐345541 suppresses breast tumorigenesis and metastases by targeting GD2 + cancer stem cells. Oncotarget 8, 36936–36949. 28415808 10.18632/oncotarget.16294 PMC5514883

[mol212557-bib-0005] Bjerkvig R , Tysnes BB , Aboody KS , Najbauer J and Terzis AJ (2005) Opinion: the origin of the cancer stem cell: current controversies and new insights. Nat Rev Cancer 5, 899–904. 16327766 10.1038/nrc1740

[mol212557-bib-0006] Bonuccelli G , Peiris‐Pages M , Ozsvari B , Martinez‐Outschoorn UE , Sotgia F and Lisanti MP (2017) Targeting cancer stem cell propagation with palbociclib, a CDK4/6 inhibitor: telomerase drives tumor cell heterogeneity. Oncotarget 8, 9868–9884. 28039467 10.18632/oncotarget.14196 PMC5354777

[mol212557-bib-0007] Bozok Cetintas V , Acikgoz E , Yigitturk G , Demir K , Oktem G , Tezcanli Kaymaz B , Oltulu F and Aktug H (2016) Effects of flavopiridol on critical regulation pathways of CD133high/CD44high lung cancer stem cells. Medicine 95, e5150. 27787370 10.1097/MD.0000000000005150 PMC5089099

[mol212557-bib-0008] Brun SN , Markant SL , Esparza LA , Garcia G , Terry D , Huang JM , Pavlyukov MS , Li XN , Grant GA , Crawford JR *et al* (2015) Survivin as a therapeutic target in Sonic hedgehog‐driven medulloblastoma. Oncogene 34, 3770–3779. 25241898 10.1038/onc.2014.304 PMC4369477

[mol212557-bib-0009] Capper D , Stichel D , Sahm F , Jones D , Schrimpf D , Sill M , Schmid S , Hovestadt V , Reuss DE , Koelsche C *et al* (2018) Practical implementation of DNA methylation and copy‐number‐based CNS tumor diagnostics: the Heidelberg experience. Acta Neuropathol 136, 181–210. 29967940 10.1007/s00401-018-1879-y PMC6060790

[mol212557-bib-0010] Cavalli F , Remke M , Rampasek L , Peacock J , Shih D , Luu B , Garzia L , Torchia J , Nor C , Morrissy AS *et al* (2017) Intertumoral heterogeneity within medulloblastoma subgroups. Cancer Cell 31, 737–754.e6. 28609654 10.1016/j.ccell.2017.05.005 PMC6163053

[mol212557-bib-0011] Chen Q , Liu X , Xu L , Wang Y , Wang S , Li Q , Huang Y and Liu T (2016) Long non‐coding RNA BACE1‐AS is a novel target for anisomycin‐mediated suppression of ovarian cancer stem cell proliferation and invasion. Oncol Rep 35, 1916–1924. 26783004 10.3892/or.2016.4571

[mol212557-bib-0012] Chen J , Shao R , Li F , Monteiro M , Liu JP , Xu ZP and Gu W (2015) PI3K/Akt/mTOR pathway dual inhibitor BEZ235 suppresses the stemness of colon cancer stem cells. Clin Exp Pharmacol Physiol 42, 1317–1326. 26399781 10.1111/1440-1681.12493

[mol212557-bib-0013] Cheng CC , Chang J , Huang SC , Lin HC , Ho AS , Lim KH , Chang CC , Huang L , Chang YC , Chang YF *et al* (2017) YM155 as an inhibitor of cancer stemness simultaneously inhibits autophosphorylation of epidermal growth factor receptor and G9a‐mediated stemness in lung cancer cells. PLoS ONE 12, e0182149. 28787001 10.1371/journal.pone.0182149 PMC5546577

[mol212557-bib-0014] Crawford JR , MacDonald TJ and Packer RJ (2007) Medulloblastoma in childhood: new biological advances. Lancet Neurol 6, 1073–1085. 18031705 10.1016/S1474-4422(07)70289-2

[mol212557-bib-0015] Daily K , Ho Sui SJ , Schriml LM , Dexheimer PJ , Salomonis N , Schroll R , Bush S , Keddache M , Mayhew C , Lotia S *et al* (2017) Molecular, phenotypic, and sample‐associated data to describe pluripotent stem cell lines and derivatives. Sci Data 4, 170030. 28350385 10.1038/sdata.2017.30 PMC5369318

[mol212557-bib-0016] Diller L , Chow EJ , Gurney JG , Hudson MM , Kadin‐Lottick NS , Kawashima TI , Leisenring WM , Meacham LR , Mertens AC , Mulrooney DA *et al* (2009) Chronic disease in the Childhood Cancer Survivor Study cohort: a review of published findings. J Clin Oncol 27, 2339–2355. 19364955 10.1200/JCO.2008.21.1953 PMC2677922

[mol212557-bib-0017] Dominguez‐Gomez G , Chavez‐Blanco A , Medina‐Franco JL , Saldivar‐Gonzalez F , Flores‐Torrontegui Y , Juarez M , Díaz‐Chávez J , Gonzalez‐Fierro A and Dueñas‐González A (2018) Ivermectin as an inhibitor of cancer stem‐like cells. Mol Med Rep 17, 3397–3403. 29257278 10.3892/mmr.2017.8231

[mol212557-bib-0018] Ehrhardt M , Craveiro RB , Holst MI , Pietsch T and Dilloo D (2015) The PI3K inhibitor GDC‐0941 displays promising *in vitro* and *in vivo* efficacy for targeted medulloblastoma therapy. Oncotarget 6, 802–813. 25596739 10.18632/oncotarget.2742 PMC4359256

[mol212557-bib-0019] Eppert K , Takenaka K , Lechman ER , Waldron L , Nilsson B , van Galen P , Metzeler KH , Poeppl A , Ling V , Beyene J *et al* (2011) Stem cell gene expression programs influence clinical outcome in human leukemia. Nat Med 17, 1086–1093. 21873988 10.1038/nm.2415

[mol212557-bib-0020] Fan X and Eberhart CG (2008) Medulloblastoma stem cells. J Clin Oncol 26, 2821–2827. 18539960 10.1200/JCO.2007.15.2264 PMC4508659

[mol212557-bib-0021] Fernandez AF , Assenov Y , Martin‐Subero JI , Balint B , Siebert R , Taniguchi H , Yamamoto H , Hidalgo M , Tan AC , Galm O *et al* (2012) A DNA methylation fingerprint of 1628 human samples. Genome Res 22, 407–419. 21613409 10.1101/gr.119867.110 PMC3266047

[mol212557-bib-0022] Garulli C , Kalogris C , Pietrella L , Bartolacci C , Andreani C , Falconi M , Marchini C and Amici A (2014) Dorsomorphin reverses the mesenchymal phenotype of breast cancer initiating cells by inhibition of bone morphogenetic protein signaling. Cell Signal 26, 352–362. 24280125 10.1016/j.cellsig.2013.11.022

[mol212557-bib-0023] Gentles AJ , Newman AM , Liu CL , Bratman SV , Feng W , Kim D , Nair VS , Xu Y , Khuong A , Hoang CD *et al* (2015) The prognostic landscape of genes and infiltrating immune cells across human cancers. Nat Med 21, 938–945. 26193342 10.1038/nm.3909 PMC4852857

[mol212557-bib-0024] Gibson P , Tong Y , Robinson G , Thompson MC , Currle DS , Eden C , Kranenburg TA , Hogg T , Poppleton H , Martin J *et al* (2010) Subtypes of medulloblastoma have distinct developmental origins. Nature 468, 1095–1099. 21150899 10.1038/nature09587 PMC3059767

[mol212557-bib-0025] Hong Z , Xiao M , Yang Y , Han Z , Cao Y , Li C , Wu Y , Gong Q , Zhou X , Xu D *et al* (2011) Arsenic disulfide synergizes with the phosphoinositide 3‐kinase inhibitor PI‐103 to eradicate acute myeloid leukemia stem cells by inducing differentiation. Carcinogenesis 32, 1550–1558. 21803735 10.1093/carcin/bgr176

[mol212557-bib-0026] Hou GX , Liu PP , Zhang S , Yang M , Liao J , Yang J , Hu Y , Jiang WQ , Wen S and Huang P (2018) Elimination of stem‐like cancer cell side‐population by auranofin through modulation of ROS and glycolysis. Cell Death Dis 9, 89. 29367724 10.1038/s41419-017-0159-4 PMC5833411

[mol212557-bib-0027] Hovestadt V , Jones DT , Picelli S , Wang W , Kool M , Northcott PA , Sultan M , Stachurski K , Ryzhova M , Warnatz HJ *et al* (2014) Decoding the regulatory landscape of medulloblastoma using DNA methylation sequencing. Nature 510, 537–541. 24847876 10.1038/nature13268

[mol212557-bib-0028] Hu H , Hang JJ , Han T , Zhuo M , Jiao F and Wang LW (2016) The M2 phenotype of tumor‐associated macrophages in the stroma confers a poor prognosis in pancreatic cancer. Tumour Biol 37, 8657–8664. 26738860 10.1007/s13277-015-4741-z

[mol212557-bib-0029] Jensen TO , Schmidt H , Møller HJ , Høyer M , Maniecki MB , Sjoegren P , Christensen IJ and Steiniche T (2009) Macrophage markers in serum and tumor have prognostic impact in American Joint Committee on Cancer stage I/II melanoma. J Clin Oncol 27, 3330–3337. 19528371 10.1200/JCO.2008.19.9919

[mol212557-bib-0030] Kawachi A , Yoshida H , Kitano S , Ino Y , Kato T and Hiraoka N (2018) Tumor‐associated CD204 + M2 macrophages are unfavorable prognostic indicators in uterine cervical adenocarcinoma. Cancer Sci 109, 863–870. 29274107 10.1111/cas.13476 PMC5834786

[mol212557-bib-0031] Lee C , Lee J , Choi SA , Kim SK , Wang KC , Park SH , Kim SH , Lee JY and Phi JH (2018) M1 macrophage recruitment correlates with worse outcome in SHH Medulloblastomas. BMC Cancer 18, 535. 29739450 10.1186/s12885-018-4457-8 PMC5941618

[mol212557-bib-0032] Li B , Fei DL , Flaveny CA , Dahmane N , Baubet V , Wang Z , Bai F , Pei XH , Rodriguez‐Blanco J , Hang B *et al* (2014) Pyrvinium attenuates Hedgehog signaling downstream of smoothened. Can Res 74, 4811–4821. 10.1158/0008-5472.CAN-14-0317 PMC4321822 24994715

[mol212557-bib-0033] Lin CY , Erkek S , Tong Y , Yin L , Federation AJ , Zapatka M , Haldipur P , Kawauchi D , Risch T , Warnatz HJ *et al* (2016) Active medulloblastoma enhancers reveal subgroup‐specific cellular origins. Nature 530, 57–62. 26814967 10.1038/nature16546 PMC5168934

[mol212557-bib-0034] Liu R , Wang X , Chen GY , Dalerba P , Gurney A , Hoey T , Sherlock G , Lewicki J , Shedden K and Clarke MF (2007) The prognostic role of a gene signature from tumorigenic breast‐cancer cells. N Engl J Med 356, 217–226. 17229949 10.1056/NEJMoa063994

[mol212557-bib-0035] Louis DN , Perry A , Reifenberger G , von Deimling A , Figarella‐Branger D , Cavenee WK , Ohgaki H , Wiestler OD , Kleihues P and Ellison DW (2016) The 2016 World Health Organization classification of tumors of the central nervous system: a summary. Acta Neuropathol 131, 803–820. 27157931 10.1007/s00401-016-1545-1

[mol212557-bib-0036] Ma J , Liu L , Che G , Yu N , Dai F and You Z (2010) The M1 form of tumor‐associated macrophages in non‐small cell lung cancer is positively associated with survival time. BMC Cancer 10, 112. 20338029 10.1186/1471-2407-10-112 PMC2851690

[mol212557-bib-0037] Majzner RG , Simon JS , Grosso JF , Martinez D , Pawel BR , Santi M , Merchant MS , Geoerger B , Hezam I , Marty V *et al* (2017) Assessment of programmed death‐ligand 1 expression and tumor‐associated immune cells in pediatric cancer tissues. Cancer 123, 3807–3815. 28608950 10.1002/cncr.30724

[mol212557-bib-0038] Maksimovic J , Gordon L and Oshlack A (2012) SWAN: subset‐quantile within array normalization for illumina infinium HumanMethylation450 BeadChips. Genome Biol 13, R44. 22703947 10.1186/gb-2012-13-6-r44 PMC3446316

[mol212557-bib-0039] Malkomes P , Lunger I , Luetticke A , Oppermann E , Haetscher N , Serve H , Holzer K , Bechstein WO and Rieger MA (2016) Selective AKT inhibition by MK‐2206 represses colorectal cancer‐initiating stem cells. Ann Surg Oncol 23, 2849–2857. 27059026 10.1245/s10434-016-5218-z PMC4972858

[mol212557-bib-0040] Malta TM , Sokolov A , Gentles AJ , Burzykowski T , Poisson L , Weinstein JN , Kamińska B , Huelsken J , Omberg L , Gevaert O *et al* (2018) Machine learning identifies stemness features associated with oncogenic dedifferentiation. Cell 173, 338–354.e15. 29625051 10.1016/j.cell.2018.03.034 PMC5902191

[mol212557-bib-0041] Martinez FO and Gordon S (2014) The M1 and M2 paradigm of macrophage activation: time for reassessment. *F1000Prime* . Rep 6, 13. 10.12703/P6-13 PMC3944738 24669294

[mol212557-bib-0042] Medrek C , Pontén F , Jirström K and Leandersson K (2012) The presence of tumor associated macrophages in tumor stroma as a prognostic marker for breast cancer patients. BMC Cancer 12, 306. 22824040 10.1186/1471-2407-12-306 PMC3414782

[mol212557-bib-0043] Mei J , Xiao Z , Guo C , Pu Q , Ma L , Liu C , Lin F , Liao H , You Z and Liu L (2016) Prognostic impact of tumor‐associated macrophage infiltration in non‐small cell lung cancer: a systemic review and meta‐analysis. Oncotarget 7, 34217–34228. 27144518 10.18632/oncotarget.9079 PMC5085150

[mol212557-bib-0044] Merlos‐Suárez A , Barriga FM , Jung P , Iglesias M , Céspedes MV , Rossell D , Sevillano M , Hernando‐Momblona X , da Silva‐Diz V , Muñoz P *et al* (2011) The intestinal stem cell signature identifies colorectal cancer stem cells and predicts disease relapse. Cell Stem Cell 8, 511–524. 21419747 10.1016/j.stem.2011.02.020

[mol212557-bib-0045] Morrissy AS , Garzia L , Shih DJ , Zuyderduyn S , Huang X , Skowron P , Remke M , Cavalli FM , Ramaswamy V , Lindsay PE *et al* (2016) Divergent clonal selection dominates medulloblastoma at recurrence. Nature 529, 351–357. 26760213 10.1038/nature16478 PMC4936195

[mol212557-bib-0046] Northcott PA , Jones DT , Kool M , Robinson GW , Gilbertson RJ , Cho YJ , Pomeroy SL , Korshunov A , Lichter P , Taylor MD *et al* (2012) Medulloblastomics: the end of the beginning. Nat Rev Cancer 12, 818–834. 23175120 10.1038/nrc3410 PMC3889646

[mol212557-bib-0047] Packer RJ and Vezina G (2008) Management of and prognosis with medulloblastoma: therapy at a crossroads. Arch Neurol 65, 1419–1424. 19001159 10.1001/archneur.65.11.1419

[mol212557-bib-0048] Packer RJ , Zhou T , Holmes E , Vezina G and Gajjar A (2013) Survival and secondary tumors in children with medulloblastoma receiving radiotherapy and adjuvant chemotherapy: results of Children's Oncology Group trial A9961. Neuro Oncol 15, 97–103. 23099653 10.1093/neuonc/nos267 PMC3534419

[mol212557-bib-0049] Ramaswamy V , Remke M , Adamski J , Bartels U , Tabori U , Wang X , Huang A , Hawkins C , Mabbott D , Laperriere N *et al* (2016a) Medulloblastoma subgroup‐specific outcomes in irradiated children: who are the true high‐risk patients. Neuro Oncol 18, 291–297. 25605817 10.1093/neuonc/nou357 PMC4724171

[mol212557-bib-0050] Ramaswamy V , Remke M , Bouffet E , Bailey S , Clifford SC , Doz F , Kool M , Dufour C , Vassal G , Milde T *et al* (2016b) Risk stratification of childhood medulloblastoma in the molecular era: the current consensus. Acta Neuropathol 131, 821–831. 27040285 10.1007/s00401-016-1569-6 PMC4867119

[mol212557-bib-0051] Ramaswamy V , Remke M , Bouffet E , Faria CC , Perreault S , Cho YJ , Shih DJ , Luu B , Dubuc AM , Northcott PA *et al* (2013) Recurrence patterns across medulloblastoma subgroups: an integrated clinical and molecular analysis. Lancet Oncol 14, 1200–1207. 24140199 10.1016/S1470-2045(13)70449-2 PMC3953419

[mol212557-bib-0052] Ramaswamy V and Taylor MD (2017) Medulloblastoma: from myth to molecular. J Clin Oncol 35, 2355–2363. 28640708 10.1200/JCO.2017.72.7842

[mol212557-bib-0053] Read TA , Fogarty MP , Markant SL , McLendon RE , Wei Z , Ellison DW , Febbo PG and Wechsler‐Reya RJ (2009) Identification of CD15 as a marker for tumor‐propagating cells in a mouse model of medulloblastoma. Cancer Cell 15, 135–147. 19185848 10.1016/j.ccr.2008.12.016 PMC2664097

[mol212557-bib-0054] Ritchie ME , Phipson B , Wu D , Hu Y , Law CW , Shi W and Smyth GK (2015) limma powers differential expression analyses for RNA‐sequencing and microarray studies. Nucleic Acids Res 43, e47. 25605792 10.1093/nar/gkv007 PMC4402510

[mol212557-bib-0055] Salomonis N , Dexheimer PJ , Omberg L , Schroll R , Bush S , Huo J , Schriml L , Ho Sui S , Keddache M , Mayhew C *et al* (2016) Integrated genomic analysis of diverse induced pluripotent stem cells from the progenitor cell biology consortium. Stem Cell Reports 7, 110–125. 27293150 10.1016/j.stemcr.2016.05.006 PMC4944587

[mol212557-bib-0056] Schüller U , Heine VM , Mao J , Kho AT , Dillon AK , Han YG , Huillard E , Sun T , Ligon AH , Qian Y *et al* (2008) Acquisition of granule neuron precursor identity is a critical determinant of progenitor cell competence to form Shh‐induced medulloblastoma. Cancer Cell 14, 123–134. 18691547 10.1016/j.ccr.2008.07.005 PMC2597270

[mol212557-bib-0057] Singh SK , Hawkins C , Clarke ID , Squire JA , Bayani J , Hide T , Henkelman RM , Cusimano MD and Dirks PB (2004) Identification of human brain tumour initiating cells. Nature 432, 396–401. 15549107 10.1038/nature03128

[mol212557-bib-0058] Sokolov A , Paull EO and Stuart JM (2016) One‐class detection of cell states in tumor subtypes. Pac Symp Biocomput 21, 405–416. 26776204 PMC4856035

[mol212557-bib-0059] Subramanian A , Narayan R , Corsello SM , Peck DD , Natoli TE , Lu X , Gould J , Davis JF , Tubelli AA , Asiedu JK *et al* (2017) A next generation connectivity map: L1000 platform and the first 1,000,000 profiles. Cell 171, 1437–1452.e17. 29195078 10.1016/j.cell.2017.10.049 PMC5990023

[mol212557-bib-0060] Taylor MD , Northcott PA , Korshunov A , Remke M , Cho YJ , Clifford SC , Eberhart CG , Parsons DW , Rutkowski S , Gajjar A *et al* (2012) Molecular subgroups of medulloblastoma: the current consensus. Acta Neuropathol 123, 465–472. 22134537 10.1007/s00401-011-0922-z PMC3306779

[mol212557-bib-0061] Thorsson V , Gibbs DL , Brown SD , Wolf D , Bortone DS , Ou Yang TH , Porta‐Pardo E , Gao GF , Plaisier CL , Eddy JA *et al* (2018) The immune landscape of cancer. Immunity 48, 812–830.e14. 29628290 10.1016/j.immuni.2018.03.023 PMC5982584

[mol212557-bib-0062] Tibshirani R (1997) The lasso method for variable selection in the Cox model. Stat Med 16, 385–395. 9044528 10.1002/(sici)1097-0258(19970228)16:4<385::aid-sim380>3.0.co;2-3

[mol212557-bib-0063] Van Overmeire E , Laoui D , Keirsse J , Van Ginderachter JA and Sarukhan A (2014) Mechanisms driving macrophage diversity and specialization in distinct tumor microenvironments and parallelisms with other tissues. Front Immunol 5, 127. 24723924 10.3389/fimmu.2014.00127 PMC3972476

[mol212557-bib-0064] Vanner RJ , Remke M , Gallo M , Selvadurai HJ , Coutinho F , Lee L , Kushida M , Head R , Morrissy S , Zhu X *et al* (2014) Quiescent sox2(+) cells drive hierarchical growth and relapse in sonic hedgehog subgroup medulloblastoma. Cancer Cell 26, 33–47. 24954133 10.1016/j.ccr.2014.05.005 PMC4441014

[mol212557-bib-0065] Vermeulen JF , Van Hecke W , Adriaansen E , Jansen MK , Bouma RG , Villacorta Hidalgo J , Fisch P , Broekhuizen R , Spliet W , Kool M *et al* (2018) Prognostic relevance of tumor‐infiltrating lymphocytes and immune checkpoints in pediatric medulloblastoma. Oncoimmunology 7, e1398877. 29399402 10.1080/2162402X.2017.1398877 PMC5790383

[mol212557-bib-0066] Ward RJ , Lee L , Graham K , Satkunendran T , Yoshikawa K , Ling E , Harper L , Austin R , Nieuwenhuis E , Clarke ID *et al* (2009) Multipotent CD15 + cancer stem cells in patched‐1‐deficient mouse medulloblastoma. Can Res 69, 4682–4690. 10.1158/0008-5472.CAN-09-0342 19487286

[mol212557-bib-0067] Xiang D , Shigdar S , Bean AG , Bruce M , Yang W , Mathesh M , Wang T , Yin W , Tran PH , Al Shamaileh H *et al* (2017) Transforming doxorubicin into a cancer stem cell killer via EpCAM aptamer‐mediated delivery. Theranostics 7, 4071–4086. 29158811 10.7150/thno.20168 PMC5694998

[mol212557-bib-0068] Xu L , Zhang L , Hu C , Liang S , Fei X , Yan N , Zhang Y and Zhang F (2016) WNT pathway inhibitor pyrvinium pamoate inhibits the self‐renewal and metastasis of breast cancer stem cells. Int J Oncol 48, 1175–1186. 26781188 10.3892/ijo.2016.3337

[mol212557-bib-0069] Yang ZJ , Ellis T , Markant SL , Read TA , Kessler JD , Bourboulas M , Schüller U , Machold R , Fishell G , Rowitch DH *et al* (2008) Medulloblastoma can be initiated by deletion of Patched in lineage‐restricted progenitors or stem cells. Cancer Cell 14, 135–145. 18691548 10.1016/j.ccr.2008.07.003 PMC2538687

[mol212557-bib-0070] Yeh CT , Su CL , Huang CY , Lin JK , Lee WH , Chang PM , Kuo YL , Liu YW , Wang LS , Wu CH *et al* (2013) A preclinical evaluation of antimycin a as a potential antilung cancer stem cell agent. Evid Based Complement Alternat Med 2013, 910451. 23840269 10.1155/2013/910451 PMC3693105

[mol212557-bib-0071] Yin Y , Liu L , Zhao Z , Yin L , Bauer N , Nwaeburu CC , Gladkich J , Gross W , Hackert T , Sticht C *et al* (2018) Simvastatin inhibits sonic hedgehog signaling and stemness features of pancreatic cancer. Cancer Lett 426, 14–24. 29627496 10.1016/j.canlet.2018.04.001

[mol212557-bib-0072] Yoshihara K , Shahmoradgoli M , Martínez E , Vegesna R , Kim H , Torres‐Garcia W , Treviño V , Shen H , Laird PW , Levine DA *et al* (2013) Inferring tumour purity and stromal and immune cell admixture from expression data. Nat Commun 4, 2612. 24113773 10.1038/ncomms3612 PMC3826632

[mol212557-bib-0073] You JS , Kang JK , Seo DW , Park JH , Park JW , Lee JC , Jeon YJ , Cho EJ and Han JW (2009) Depletion of embryonic stem cell signature by histone deacetylase inhibitor in NCCIT cells: involvement of Nanog suppression. Can Res 69, 5716–5725. 10.1158/0008-5472.CAN-08-4953 19567677

[mol212557-bib-0074] Zhang H , Li H , Liu Z , Ge A , Guo E , Liu S and Chen Z (2018) Triptolide inhibits the proliferation and migration of medulloblastoma Daoy cells by upregulation of microRNA‐138. J Cell Biochem 119, 9866–9877. 30156009 10.1002/jcb.27307

[mol212557-bib-0075] Zhang FL , Wang P , Liu YH , Liu LB , Liu XB , Li Z and Xue YX (2013) Topoisomerase I inhibitors, shikonin and topotecan, inhibit growth and induce apoptosis of glioma cells and glioma stem cells. PLoS ONE 8, e81815. 24303074 10.1371/journal.pone.0081815 PMC3841142

[mol212557-bib-0076] Zheng Y , de la Cruz CC , Sayles LC , Alleyne‐Chin C , Vaka D , Knaak TD , Bigos M , Xu Y , Hoang CD , Shrager JB *et al* (2013a) A rare population of CD24(+)ITGB4(+)Notch(hi) cells drives tumor propagation in NSCLC and requires Notch3 for self‐renewal. Cancer Cell 24, 59–74. 23845442 10.1016/j.ccr.2013.05.021 PMC3923526

[mol212557-bib-0077] Zheng X , Naiditch J , Czurylo M , Jie C , Lautz T , Clark S , Jafari N , Qiu Y , Chu F and Madonna MB (2013b) Differential effect of long‐term drug selection with doxorubicin and vorinostat on neuroblastoma cells with cancer stem cell characteristics. Cell Death Dis 4, e740. 23887631 10.1038/cddis.2013.264 PMC3730434

