# Supplementary material for: Integrative analysis of gene expression and DNA methylation through one‐class logistic regression machine learning identifies stemness features in medulloblastoma
Source: Mol Oncol. 2019 Aug 18;13(10):2227–45. doi: 10.1002/1878-0261.12557 (PMC6763787; doi:10.1002/1878-0261.12557)
Supplement: Supplementary file 1 — Table S1. Clinicopathological features of patients in the GSE85218 dataset. Table S2. Comparison of distribution of clinical characteristics between the training and validation set. Table S3. The multivariate Cox regression coefficients of the genes in the 23‐mRNA‐based prognostic model. Table S4. Comparisons of the predictive value of the 23‐mRNA‐based prognostic model and the random model based on a random subset of 23 genes. Table S5. Compounds with an enrichment score ≤ −95 that could target pathways associated with MB stemness. [file MOL2-13-2227-s001.docx]

**Table S1.** Clinicopathological features of patients in the GSE85218 dataset.

|  |  | **Number** | **Percentage, %** |
| --- | --- | --- | --- |
| **Total** |  | 763 | 100.00 |
| **Age, years** |  | 0.2-56.8 (8) |  |
|  | < median | 340 | 44.56 |
|  | ≥ median | 389 | 50.98 |
|  | NA | 34 | 4.46 |
| **Gender** |  |  |  |
|  | Female | 247 | 32.37 |
|  | Male | 472 | 61.86 |
|  | NA | 44 | 5.77 |
| **Histology** |  |  |  |
|  | Classic | 387 | 50.72 |
|  | Desmoplastic | 109 | 14.29 |
|  | LC/A | 72 | 9.44 |
|  | MBEN | 18 | 2.36 |
|  | NA | 177 | 23.19 |
| **Metastatic status** | |  |  |
|  | non-metastatic | 397 | 52.03 |
|  | Metastatic | 176 | 23.07 |
|  | NA | 190 | 24.90 |
| **Subgroup** |  |  |  |
|  | WNT | 70 | 9.17 |
|  | SHH | 223 | 29.23 |
|  | Group 3 | 144 | 18.87 |
|  | Group 4 | 326 | 42.73 |

Abbreviations: LC/A, large cell/anaplastic;

MBEN, medulloblastoma with extensive nodularity;

NA, not applicable.

**Table S2.** Comparison of distribution of clinical characteristics between the training and validation set.

| **Parameter** | **Training set**  (n= 121) | **Validation set**  (n=51) | p-value* |
| --- | --- | --- | --- |
| **Age** (median, IQR) | 8.1 (2.5-21.8) | 7.2 (2.1-15.2) | 0.24 |
| **Gender** (n, %) | | | |
| Female | 46 (39.0) | 17 (34.0) | 0.66 |
| Male | 72 (61.0) | 33 (66.0) |  |
| **Histology** (n, %) | | | |
| Classic | 44 (43.1) | 20 (46.5) | 0.21 |
| Desmoplastic | 44 (43.1) | 12 (27.9) |  |
| LC/A | 10 (9.8) | 7 (16.3) |  |
| MBEN | 4 (3.9) | 4 (9.3) |  |
| **Metastatic status** (n, %) |  |  |  |
| Non-metastatic | 84 (84.0) | 40 (87.0) | 0.83 |
| Metastatic | 16 (16.0) | 6 (13.0) |  |

Annotations: *group differences evaluated with chi-square test for categorical parameters and Kruskal-Wallis test for continuous parameters.

Abbreviations: IQR, interquartile range; LC/A, large cell/anaplastic; MBEN, medulloblastoma with extensive nodularity.

**Table S3.** The multivariate Cox regression coefficients of the genes in the 23-mRNA-based prognostic model.

| **Gene name** | **Coefficient** |
| --- | --- |
| ADAMTSL3 | -0.140812 |
| COLGALT1 | 0.901308 |
| CPE | -0.826426 |
| EFEMP2 | -0.409694 |
| FAM214A | -0.172157 |
| FKBP4 | -0.277554 |
| FRZB | -0.131879 |
| HIST1H2APS4 | -0.303753 |
| ITIH2 | -0.0985019 |
| KCNG1 | -0.220376 |
| KIAA0825 | 0.248612 |
| LDB3 | 0.121513 |
| LPCAT3 | -0.281309 |
| MTRR | -0.266869 |
| NLGN4Y | -0.564694 |
| PIP4K2A | 1.55179 |
| PROSER1 | 0.0238936 |
| TIMM50 | -0.5312 |
| TMEM185B | 0.75654 |
| TMEM38B | 1.35218 |
| TOMM40 | 0.926504 |
| TRIM28 | 0.774871 |
| TRMT1 | 2.06407 |

**Table S4.** Comparisons of the predictive value of the 23-mRNA-based prognostic model and the random model based on a random subset of 23 genes.

|  | The random model |  | The 23-mRNA-based model |  |
| --- | --- | --- | --- | --- |
| Dataset | AUC (95%, CI) |  | AUC | *P** |
| Patients having the 1‐year OS in the training set | 0.804 (0.798-0.810) |  | 0.769 | 1 |
| Patients having the 3‐year OS in the training set | 0.832 (0.828-0.835) |  | 0.842 | 8.81E-09 |
| Patients having the 5‐year OS in the training set | 0.836 (0.833-0.840) |  | 0.862 | <2.2e-16 |
| Patients having the 1‐year OS in the validation set | 0.639 (0.630-0.649) |  | 0.827 | <2.2e-16 |
| Patients having the 3‐year OS in the validation set | 0.583 (0.578-0.588) |  | 0.763 | <2.2e-16 |
| Patients having the 5‐year OS in the validation set | 0.610 (0.605-0.616) |  | 0.753 | <2.2e-16 |

*Student's t-test was used for comparison of AUC values of the two models.

Abbreviations: OS, overall survival; AUC, area under the curve.

**Table S5.** Compounds with an enrichment score ≤ -95 that could target pathways associated with MB stemness.

| Score | Compound | Mechanisms of action |
| --- | --- | --- |
| -99.86 | palbociclib | CDK inhibitor |
| -99.65 | aminopurvalanol-a | Tyrosine kinase inhibitor |
| -99.47 | purvalanol-a | CDK inhibitor |
| -99.37 | bisbenzimide | DNA binding agent |
| -99.36 | JAK3-inhibitor-VI | JAK inhibitor |
| -98.94 | CAY-10470 | NFkB pathway inhibitor |
| -98.84 | BX-795 | IKK inhibitor |
| -98.77 | droxinostat | HDAC inhibitor |
| -98.77 | AZD-8055 | MTOR inhibitor |
| -98.66 | camptothecin | Topoisomerase inhibitor |
| -98.59 | dorsomorphin | AMPK inhibitor |
| -98.56 | amonafide | Topoisomerase inhibitor |
| -98.52 | wortmannin | PI3K inhibitor |
| -98.48 | PHA-793887 | CDK inhibitor |
| -98.41 | NCH-51 | HDAC inhibitor |
| -98.34 | ZG-10 | JNK inhibitor |
| -98.34 | JNJ-7706621 | CDK inhibitor |
| -98.34 | bisindolylmaleimide-ix | CDK inhibitor |
| -98.34 | BMS-345541 | IKK inhibitor |
| -98.31 | AS-601245 | JNK inhibitor |
| -98.27 | angiogenesis-inhibitor | Angiogenesis inhibitor |
| -98.27 | cyclopamine | Smoothened receptor antagonist |
| -98.24 | Merck60 | HDAC inhibitor |
| -98.24 | chromomycin-a3 | DNA binding agent |
| -98.24 | PF-562271 | Focal adhesion kinase inhibitor |
| -98.2 | CGP-60474 | CDK inhibitor |
| -98.2 | JNK-9L | JNK inhibitor |
| -98.2 | AT-7519 | CDK inhibitor |
| -98.17 | topotecan | Topoisomerase inhibitor |
| -98.13 | PI-103 | MTOR inhibitor |
| -98.06 | A-443644 | AKT inhibitor |
| -98.06 | PIK-75 | DNA protein kinase inhibitor |
| -98.06 | ivermectin | GABA receptor agonist |
| -97.96 | BMS-754807 | IGF-1 inhibitor |
| -97.96 | lestaurtinib | FLT3 inhibitor |
| -97.95 | doxorubicin | Topoisomerase inhibitor |
| -97.92 | pidorubicine | Topoisomerase inhibitor |
| -97.92 | ER-27319 | Mediator release inhibitor |
| -97.89 | WYE-125132 | MTOR inhibitor |
| -97.86 | indolophenanthridine | CALY activator |
| -97.82 | auranofin | NFkB pathway inhibitor |
| -97.78 | dactolisib | MTOR inhibitor |
| -97.75 | RO-08-2750 | NGF binding inhibitor |
| -97.64 | YM-155 | Survivin inhibitor |
| -97.6 | pyrvinium-pamoate | AKT inhibitor |
| -97.6 | WYE-354 | MTOR inhibitor |
| -97.53 | mitoxantrone | Topoisomerase inhibitor |
| -97.29 | alvocidib | CDK inhibitor |
| -97.29 | LDN-193189 | Serine/threonine kinase inhibitor |
| -97.26 | alisertib | Aurora kinase inhibitor |
| -97.18 | dactinomycin | RNA polymerase inhibitor |
| -97.18 | SN-38 | Topoisomerase inhibitor |
| -97.16 | KU-0063794 | MTOR inhibitor |
| -97.08 | IKK-16 | IKK inhibitor |
| -97.04 | CD-437 | Retinoid receptor agonist |
| -96.96 | AG-879 | Angiogenesis inhibitor |
| -96.86 | teniposide | Topoisomerase inhibitor |
| -96.86 | pirarubicin | Topoisomerase inhibitor |
| -96.83 | manumycin-a | Farnesyltransferase inhibitor |
| -96.72 | ISOX | HDAC inhibitor |
| -96.72 | PI-828 | PI3K inhibitor |
| -96.59 | entinostat | HDAC inhibitor |
| -96.48 | 7b-cis | Exportin antagonist |
| -96.46 | VAMA-37 | DNA dependent protein kinase inhibitor |
| -96.4 | linifanib | PDGFR receptor inhibitor |
| -96.37 | tacedinaline | HDAC inhibitor |
| -96.37 | methylene-blue | Guanylyl cyclase inhibitor |
| -96.35 | etoposide | Topoisomerase inhibitor |
| -96.26 | quinoclamine | Algicide |
| -96.23 | HG-5-113-01 | Protein kinase inhibitor |
| -96.23 | SB-218078 | CHK inhibitor |
| -96.16 | vorinostat | HDAC inhibitor |
| -96.08 | cercosporin | Photoactivated toxin |
| -96.03 | WT-171 | HDAC inhibitor |
| -95.95 | GDC-0941 | PI3K inhibitor |
| -95.91 | SCH-79797 | Proteasome inhibitor |
| -95.88 | irinotecan | Topoisomerase inhibitor |
| -95.84 | APHA-compound-8 | HDAC inhibitor |
| -95.82 | daunorubicin | RNA synthesis inhibitor |
| -95.81 | PIK-90 | PI3K inhibitor |
| -95.81 | triptolide | RNA polymerase inhibitor |
| -95.77 | MK-2206 | AKT inhibitor |
| -95.75 | apicidin | HDAC inhibitor |
| -95.7 | DL-PDMP | Glucosyltransferase inhibitor |
| -95.6 | NSC-3852 | HDAC inhibitor |
| -95.5 | cyclosporin-a | Calcineurin inhibitor |
| -95.42 | HU-211 | Glutamate receptor antagonist |
| -95.39 | 5-iodotubercidin | Adenosine kinase inhibitor |
| -95.38 | JTC-801 | Opioid receptor antagonist |
| -95.26 | antimycin-a | ATP synthase inhibitor |
| -95.1 | simvastatin | HMGCR inhibitor |
| -95.1 | anisomycin | DNA synthesis inhibitor |
| -95.1 | digoxin | ATPase inhibitor |
| -95.07 | malonoben | Protein tyrosine kinase inhibitor |
| -95.05 | givinostat | HDAC inhibitor |
| -95 | mocetinostat | HDAC inhibitor |
